# Transglutaminase-Mediated Semen Coagulation Controls Sperm Storage in the Malaria Mosquito

**DOI:** 10.1371/journal.pbio.1000272

**Published:** 2009-12-22

**Authors:** David W. Rogers, Francesco Baldini, Francesca Battaglia, Maria Panico, Anne Dell, Howard R. Morris, Flaminia Catteruccia

**Affiliations:** 1 Division of Cell and Molecular Biology, Imperial College London, London, United Kingdom; 2 Università degli Studi di Perugia, Dipartimento di Medicina Sperimentale e Scienze Biochimiche, Terni, Italy; 3 Division of Molecular Biosciences, Imperial College London, London, United Kingdom; Stanford University, United States of America

## Abstract

Insect seminal fluid proteins are powerful modulators of many aspects of female physiology and behaviour including longevity, egg production, sperm storage, and remating. The crucial role of these proteins in reproduction makes them promising targets for developing tools aimed at reducing the population sizes of vectors of disease. In the malaria mosquito *Anopheles gambiae*, seminal secretions produced by the male accessory glands (MAGs) are transferred to females in the form of a coagulated mass called the mating plug. The potential of seminal fluid proteins as tools for mosquito control demands that we improve our limited understanding of the composition and function of the plug. Here, we show that the plug is a key determinant of *An. gambiae* reproductive success. We uncover the composition of the plug and demonstrate it is formed through the cross-linking of seminal proteins mediated by a MAG-specific transglutaminase (TGase), a mechanism remarkably similar to mammalian semen coagulation. Interfering with TGase expression in males inhibits plug formation and transfer, and prevents females from storing sperm with obvious consequences for fertility. Moreover, we show that the MAG-specific TGase is restricted to the anopheline lineage, where it functions to promote sperm storage rather than as a mechanical barrier to re-insemination. Taken together, these data represent a major advance in our understanding of the factors shaping *Anopheles* reproductive biology.

## Abstract 2

The mating plug is a key regulator of mosquito fertility.

## Author Summary

Male seminal fluid proteins trigger a wide range of behavioural and physiological changes in females and can have important effects on reproductive success. In many animals, seminal fluid is transferred to females as a gelatinous mass termed a mating plug. Although many hypotheses have been put forward to explain the function of mating plugs, their precise role in most organisms remains unclear. We have studied the composition, mechanism of formation, and function of the mating plug in the mosquito *Anopheles gambiae*, the principal vector of human malaria. We show that the plug is formed through the action of a transglutaminase enzyme that links seminal fluid proteins together resulting in semen coagulation. This process is similar to the way seminal fluid is coagulated in mammals. Interfering with the production of this transglutaminase prevented plug formation. Females that did not receive a plug failed to store sperm correctly, with important consequences for fertility. Our data show that the mating plug is an important feature of *An. gambiae* reproduction, and reinforce the notion that a deeper understanding of mosquito reproductive biology can aid efforts to eradicate these disease vectors.

## Introduction

*Anopheles gambiae* mosquitoes are the principal vectors of human malaria, a disease with devastating consequences for public health and the economic development of disease-endemic countries. The creation of new tools to control vector populations is a focal point of intensive efforts to eradicate the burden of malaria. As mosquitoes generally copulate only once during their lives [1], interfering with the mating process is a promising avenue for research into vector control. Unfortunately, very little is known about the molecular or physiological basis of mating and insemination in malaria vectors. Of particular concern is our lack of knowledge about factors and pathways ensuring male reproductive success, such as those that result in sperm storage, oviposition, and the inhibition of remating in females. Improving our understanding of mating biology might not only inform currently proposed strategies for vector control [2], but could potentially allow the development of entirely novel tools for combating malaria.

In sharp contrast with this scenario, a wealth of information is available on the mating biology of some non-vector insect species, particularly the fruit fly *Drosophila melanogaster*. Seminal fluid proteins (generally named Acps) derived from the male accessory glands (MAGs) and transferred to females during copulation have been demonstrated to be crucial regulators of sperm storage and viability and to be the triggers of oviposition and the reduced receptivity to remating experienced by *D. melanogaster* females after mating (reviewed in [3]). Recently, large numbers of MAG-expressed proteins have been identified in numerous insects (e.g., honeybees [4], butterflies [5], crickets [6], medflies [7]) including two mosquito vectors: *Aedes aegypti* [8] and *An. gambiae* [9]. However, assigning specific functions to these seminal fluid proteins has proved to be difficult. Even in *D. melanogaster*, where genetic tools are well developed, only a handful of the >100 secreted Acps identified in the MAGs have been functionally characterized [3]. Our current knowledge of the importance of seminal fluid proteins to mating biology in *An. gambiae* is limited, and their role in reproduction is inferred mainly by the presence of similar functional classes amongst *Anopheles* and *Drosophila* Acps [9]. For instance, in contrast to our understanding in *Drosophila* [10] and *Aedes aegypti* [8] where many Acps have been identified in mated females, not a single MAG-expressed protein has been demonstrated to be transferred to females in *An. gambiae* (but see [11]).

**Figure 1:**
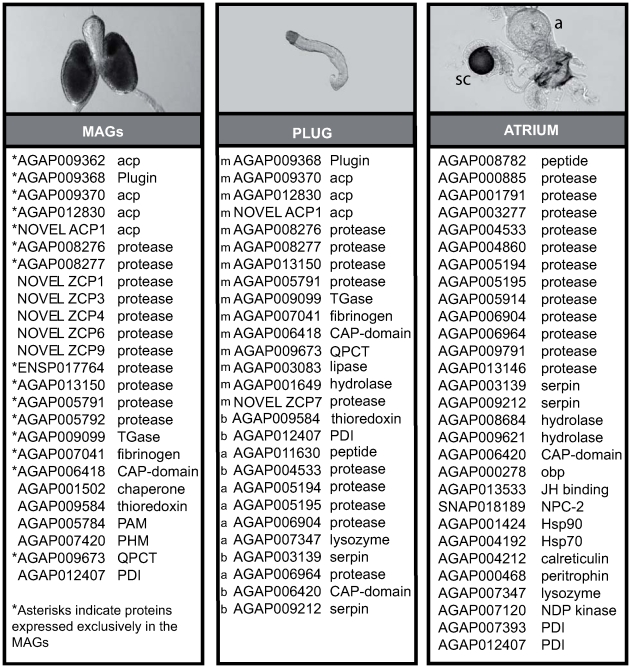
Proteins of the mating plug of *An. gambiae*. A total of 27 proteins were identified by mass spectrometry analysis of mating plugs dissected from recently mated females. The source of each protein found in the plug was determined initially by the comparative proteomic MS analysis of MAGs compared to mating plug samples compared to virgin female atrium controls, and then refined by RT-PCR. The spermathecal capsule (sc), in the figure shown next to the atrium (a), was not included in this analysis. The tissue of expression of the genes encoding the proteins found in the plug are indicated by the letters m (MAGs), a (atrium), or b (for both). Proteins are named using their VectorBase identifiers (http://www.vectorbase.org/index.php), unless currently unannotated. The novel accessory gland protein (NOVEL ACP1) is encoded by a predicted gene located between *AGAP009370* and *AGAP009371*, while the novel zinc carboxypeptidases (ZCPs) correspond to *ab initio* prediction located within the region 3R: 5051000-5067900 (see Table S3 for details). CAP, CRISP/Antigen5/PR-1; PAM, peptidylglycine α-amidating monooxygenase; PHM, peptidylglycine α-hydroxylating monooxygenase; QPCT, glutaminyl-peptide cyclotransferase; PDI, protein disulfide isomerase; obp, odorant-binding protein; JH, juvenile hormone; NPC, Nieman-Pick C; Hsp, heat-shock protein; NDP, nucleoside diphosphate.

MAG secretions in *An. gambiae* are deposited into the atrium of the female reproductive tract in the form of a gelatinous mating plug (Figure 1) [12],[13]. Mating plugs are a common feature in the reproduction of many organisms including invertebrates, reptiles, and mammals [14], however, among mosquitoes, they are exclusive to anopheline species [15]. The *An. gambiae* plug is formed entirely within the male and is digested by the female over a period of 24 h. Nothing is known about its composition or how the liquid contents of the MAGs coagulate to form a solid mass during mating. Even the function of the plug is unclear. One prominent hypothesis is that the mating plug of *An. gambiae* serves as a physical barrier to re-insemination by blocking access to the spermatheca [15],[16]. Indeed, Gillies [13] observed rare instances of females with two plugs and sperm trapped between them. Alternatively, the mating plug might act to prevent loss of sperm from the female storage organ, ensuring male reproductive success [17]. However, early researchers (e.g., [12],[18]) dismissed both of these possibilities and proposed that the mating plug serves no function and is simply a vestige of the ancestral dipteran spermatophore.

Here, we show that the plug is a crucial determinant of *An. gambiae* reproductive success. By studying its composition, we were able to identify the mechanism of plug formation, which is based on the cross-linking activity of a MAG-specific transglutaminase (TGase) on seminal proteins. RNAi-mediated depletion of this TGase in males prevents plug formation and transfer, and severely impairs fertility. Females that do not receive a mating plug cannot retain sperm in their sperm storage organ, the spermatheca, and therefore do not become inseminated. Moreover, we show that the plug provides little defence against re-insemination.

## Results

### The Mating Plug Is Composed of Multiple MAG Proteins

To identify MAG proteins that are transferred to females during copulation, we examined the composition of mating plugs dissected from the reproductive tracts of recently mated *An. gambiae* females by mass spectrometric (MS) proteomic analyses [19]–[23]. To determine the source of the proteins found in the plug, we also analyzed the composition of the MAGs, and the atria of virgin females (Figure 1 and Figure S1), followed by reverse transcription PCR (RT-PCR). These analyses identified 27 plug proteins: 15 derived from the male, six derived from the female, and six found in both male and female reproductive tissues (Figure 1). Five of the male-derived proteins (Acps) were previously shown to be exclusively expressed in the accessory glands, and included four proteins located within a “male fertilization island” on chromosome arm 3R [9]. The 10 remaining male-derived proteins in the plug were not previously known to play a role in reproduction and included five proteases. Even though proteases have been shown to be important components of the seminal fluid of other Diptera [3],[8],[10], a previous study had failed to identify these enzymes in the MAGs of *An. gambiae* [9]. The six plug proteins derived from the female reproductive tract included two secreted atrium-specific serine proteases (AGAP005194 and AGAP005195) whose transcripts were previously shown to be strongly downregulated 24 h after mating (Figure 1) [24].

**Figure 2:**
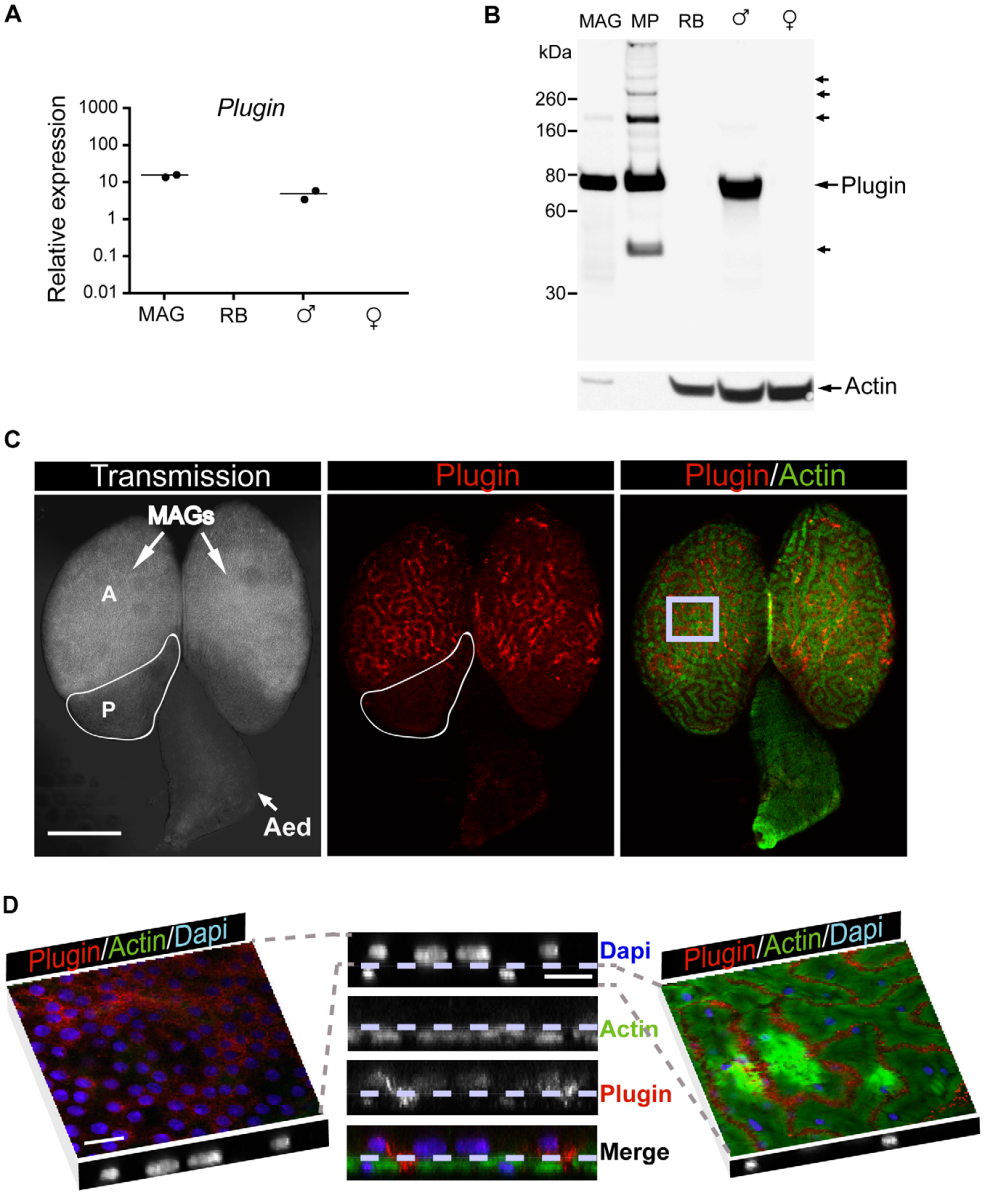
Plugin, an important component of the mating plug. Plugin protein, found mass spectrometrically in both mating plug and MAGs, is produced specifically in the MAGs. (A) qRT-PCR showing that *Plugin* is expressed specifically in the MAGs. (B) Western blot analysis of Plugin using a polyclonal antibody raised against a peptide fragment of the protein. Plugin is detected in MAGs and whole males (♂), but not in the rest of the male body (RB) or the whole female (♀). Arrows indicate the multiple Plugin immunoreactive bands observed in the mating plug (MP). Actin was used as a loading control. (C, D) Confocal analysis of Plugin expression in the MAGs of virgin 3-d-old males. Plugin (red) is concentrated in channels formed by phalloidin-Alexa 488 (green)-labelled actin-enriched muscle cells. (C) Plugin is primarily expressed in the anterior (A) rather than the posterior (P) MAGs or the aedegus (Aed). Inset in right image indicates the region analyzed in (D). Scale bar: 100 µm. (D) Central panel shows a Z-section of a 9.6 µm stack. Nuclei of epithelial (upper) and muscle (lower) cells, labelled with DAPI (blue), are clearly organized in two separate layers (highlighted by the dotted line). Scale bar: 9 µm. Left and right images represent single xy sections of upper epithelial (luminal) and lower muscular (external) layers, respectively. Scale bar: 18 µm.

Multiple gel bands in particular from 50 to 140 kDa in the MS proteomic analysis of mating plug samples contained peptides derived from one particular protein, AGAP009368, which we have named Plugin (Figure S1). Plugin was found by MS in both MAG and mating plug samples (Figure 1), and quantitative RT-PCR revealed that it is expressed exclusively in the MAGs (Figure 2A). Western blot analysis confirmed this tissue specificity and showed the presence of high molecular weight bands in plug extracts (Figure 2B). Within the MAGs, Plugin was detected by immunofluorescence primarily in the anterior region of a secretory epithelium and in the channels formed by an actin-rich muscle network lining the outside of the glands (Figure 2C, 2D).

Plugin lacks any recognizable protein domains, but is glutamine-rich (134/557 residues, Figure S1). Many of these glutamine residues are excellent candidates for TGase-mediated cross-linking sites, as they often occur in tandem with a lysine at the +2 position, and are located in a region of the protein predicted to be intrinsically disordered [25]. This observation, combined with the MS identification of both Plugin tryptic peptides in the digests of high molecular weight gel bands and a MAG-derived TGase (AGAP009099) in the mating plug, suggested that plug formation may be mediated by cross-linking of Plugin by this TGase.

### The Plug Is Formed by TGase-Mediated Cross-Linking of Plugin

**Figure 3:**
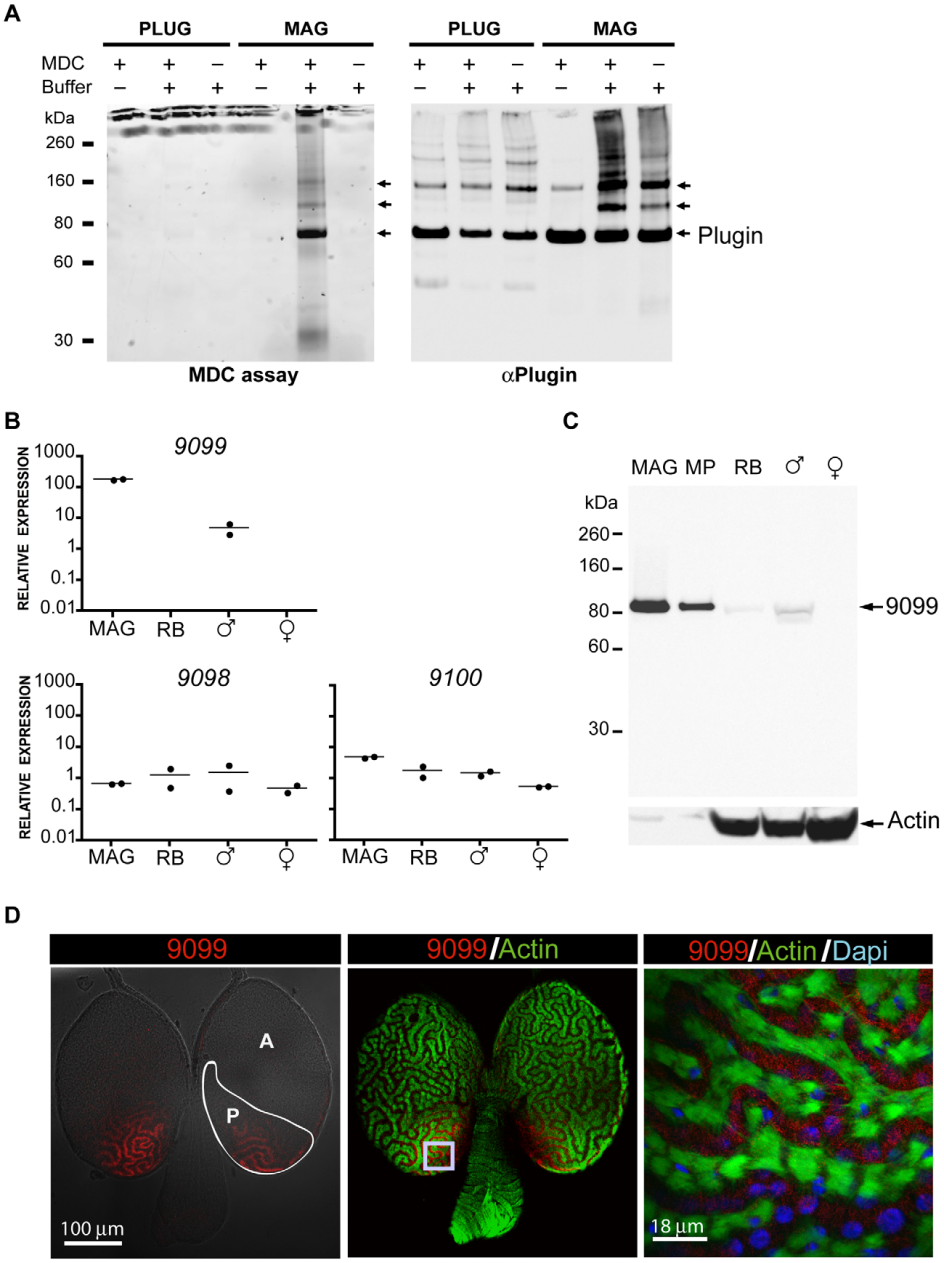
AGAP009099 is a MAG-specific TGase. AGAP009099 was found mass spectrometrically in both mating plug and MAGs. (A) MDC assay of the MAGs and plug. High levels of TGase activity in the MAGs result in the cross-linking of Plugin (high molecular weight bands), which can be prevented by TGase inhibitors (Buffer “−”). Buffer “+”: buffer promoting TGase activity. No autofluorescent proteins were detected (see the no MDC reaction). (B) qRT-PCR of *Anopheles* TGases. Upper panel: *AGAP009099* is expressed exclusively in the MAGs. No expression was detected in the male RB. Lower panel: *AGAP009098* and *AGAP009100* show low expression in all tissues tested. (C) Western blot of the MAGs using a polyclonal antibody raised against a peptide fragment of AGAP009099. AGAP009099 is also detected in mating plug (MP) and whole males (♂). Actin was used as a loading control. (D) Confocal analysis of AGAP009099 expression in the MAGs of virgin 3-d-old males. AGAP009099 (red) is concentrated in channels formed by phalloidin-Alexa 488 (green)-labelled actin-enriched muscle cells, in the posterior (P) rather than the anterior (A) MAGs. The right image is a magnification (xy section) of the region indicated by the inset in the middle figure. Cell nuclei (blue) are labelled with DAPI.

We tested for TGase activity in males using a monodansylcadaverine (MDC) incorporation assay [26], which allows the incorporation of the fluorescent amine MDC into TGase substrates to be detected under UV illumination. High levels of TGase activity were detected in homogenized MAGs, but not in the mating plug (Figure 3A), nor other male and female tissues (unpublished data). MDC was incorporated into proteins that perfectly matched the observed sizes of Plugin, strongly indicating that this protein is the primary substrate for TGase in the MAGs. This incorporation was blocked by the addition of EDTA and GTP, which suggests that the TGase activity in the MAGs is calcium-dependent. These inhibitors also greatly reduced the formation of the higher molecular weight Plugin-immunoreactive bands in the MAG samples (Figure 3A).

The high levels of TGase activity observed in the MAGs prompted a closer investigation of *An. gambiae* TGases. Unusually for insects, which are believed to possess only a single TGase [27], *An. gambiae* mosquitoes have three genes (*AGAP009098*, *AGAP009099*, and *AGAP009100*), clustered on chromosomal arm 3R. *AGAP009099* was expressed exclusively in the MAGs (Figure 3B), as confirmed by Western blot (Figure 3C), while the other two genes were ubiquitously transcribed at much lower levels (Figure 3B). These results suggested that *AGAP009099* is principally responsible for the TGase activity detected in the MAGs. Within the MAGs, the AGAP009099 protein was localized in a similar pattern to Plugin, however it was primarily concentrated in the posterior part of the glands (Figure 3D).

The role of AGAP009099 in plug formation was then tested in vivo by RNA interference-mediated knockdown. Injections of male adults with double stranded RNA (dsRNA) targeting *AGAP009099* (*ds9099*) induced a significant reduction in both transcript (mean = 67.0%, paired *t* test: *t*_11_ = −3.60, *p* = 0.0042) and protein (mean = 58.1%, *t* test assuming unequal variances: *t*_47.2_ = −4.25, *p* = 0.0001) levels relative to males injected with control dsRNA (*dsLacZ*). When injected males were allowed to mate with virgin females, 55 out of 367 females (15.0%) mated to *ds9099*-injected males failed to receive a mating plug, compared to 4 out of 228 (1.8%) females mated to control males. This large and statistically significant difference (contingency test, *χ* ^2^ = 27.55, *p*<0.0001) demonstrates that AGAP009099 is crucial for the formation of the mating plug.

### The Mating Plug Ensures Correct Sperm Storage But Is an Inefficient Barrier to Re-Insemination

We next assessed the function of the mating plug in *Anopheles* reproduction. In the large majority of cases where *ds9099*-injected males failed to transfer a mating plug, no sperm was found in the female spermatheca by microscopic analysis (41/55 = 74.5%). The absence of sperm was confirmed by our inability to amplify a Y-chromosome specific sequence by quantitative PCR in these spermathecal samples (Table S1). In these females, sperm were observed in the atria, indicating successful transfer, but were not appropriately stored and therefore would not be available for fertilization. In contrast, when a mating plug was found in the atrium, the spermatheca always contained sperm. Thus, the mating plug is important for sperm storage and for ensuring successful insemination.

Only 2.5% of field-caught female *An. gambiae* store sperm from more than one male in their spermathecae [1]. One possible explanation for the low numbers of multiple inseminations is that, prior to the establishment of long-term mating refractoriness, if females mate again within a few hours of the first copulation, the presence of a plug might effectively block sperm from the second male from entering the spermatheca. We directly tested this hypothesis by mating females with wild-type males followed in rapid succession by transgenic males to be able to distinguish alleles from this second mating. Quantitative PCR of relative quantities of the two sperm types showed that 24 of the 38 twice-mated females tested (66%) had sperm from both males in their spermathecae (mean % sperm from 2^nd^ male = 38%, range = 7%–56%), demonstrating that the mating plug is an inefficient physical barrier to re-insemination.

### A MAG-Specific TGase Is Not Present in Mosquitoes That Do Not Produce Plugs

**Figure 4:**
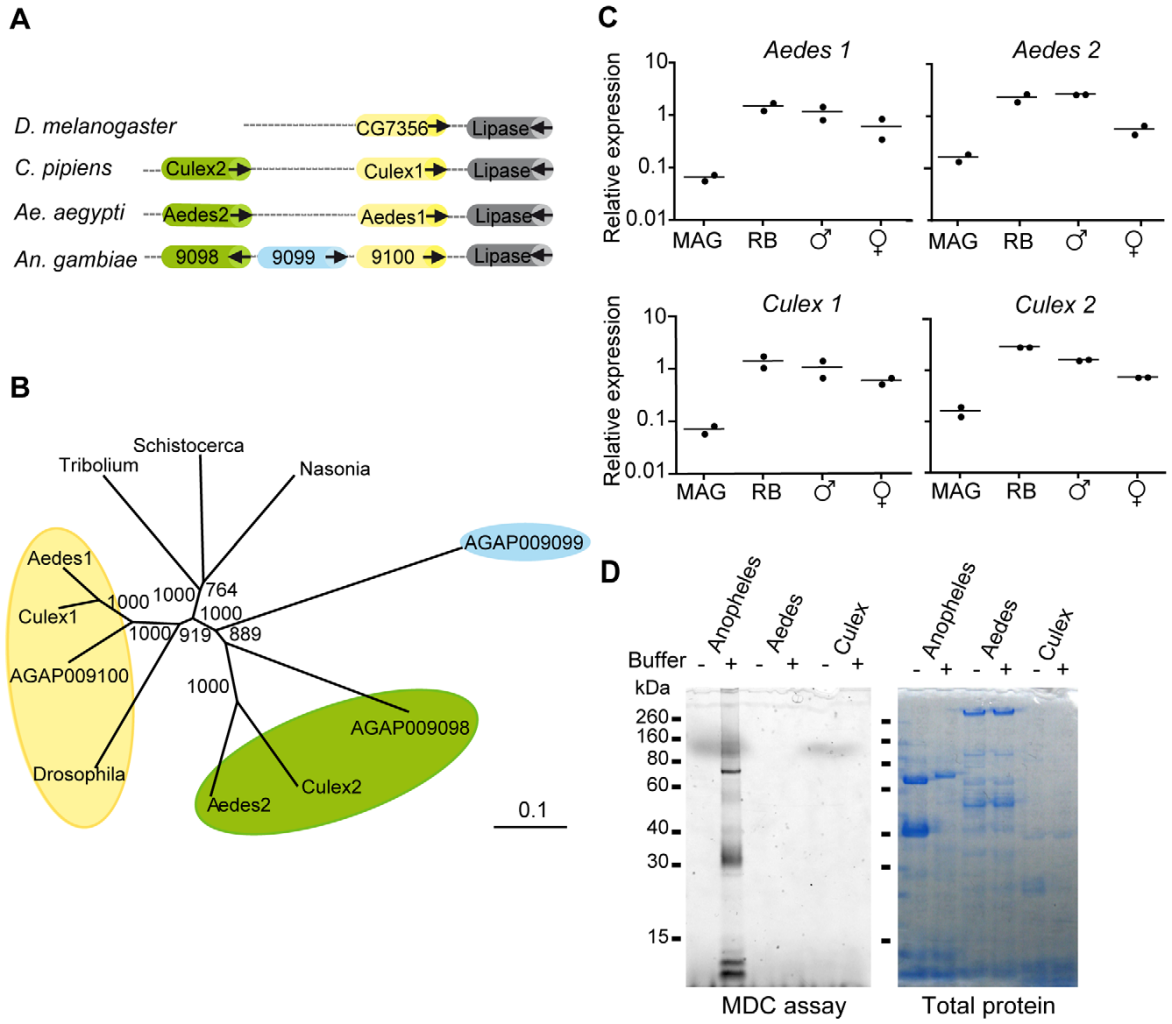
Insect species that do not make a mating plug do not possess an orthologue of AGAP009099. (A) Synteny map of Dipteran TGases. All insects included in the analysis possess one TGase (yellow) located just upstream of a conserved lipase. Mosquitoes possess a second TGase (green), while *An. gambiae* possess a third gene (blue), absent from *Ae. aegypti* and *C. quinquefasciatus*. Arrows indicate the direction of the reading frame. (B) Phylogenetic analysis of insect TGases. The bootstrap values of a 1,000 replicates are indicated. The scale bar represents the amino acid divergence. (C) qRT-PCR of culicine TGases. Neither the “ancestral” TGase (Aedes1, Culex 1) nor the mosquito-specific TGase (Aedes2, Culex2) shows high levels of expression in the MAGs. (D) TGase activity in the MAGs of three mosquito species. TGase activity was detected in the MAGs of *An. gambiae* (in Buffer “+”) but not *Ae. aegypti* or *C. quinquefasciatus*, despite the presence of protein in all samples.

Anophelines are the only mosquitoes that produce mating plugs [15]. If TGase activity underlies the ability to produce plugs, we would not expect to find a MAG-specific TGase in species that transfer uncoagulated seminal fluid, such as culicine mosquitoes. To test this hypothesis, we searched for TGase genes in the complete genomes of two culicines, *Aedes aegypti* and *Culex quinquefasciatus*, the only other mosquito species sequenced to date. We identified two culicine TGases retaining partial synteny with the three genes identified in *An. gambiae* and the single one present in *Drosophila melanogaster* (Figure 4A, Table S2). Phylogenetic analysis of TGases from these and other insects revealed that *AGAP009100*, *Aedes 1*, and *Culex 1* cluster with the single TGase from *Drosophila* (Figure 4B), suggesting that these genes may retain the ancestral function. *AGAP009098* clusters with the second culicine TGase (*Aedes 2* and *Culex 2*) in a mosquito-specific group. No culicine TGase clusters with *AGAP009099*, consistent with the lack of seminal coagulation in these mosquitoes. Importantly, neither of the *Aedes* or *Culex* TGase genes showed enriched expression in the MAGs (Figure 4C), and we found no evidence of TGase activity in the glands of either species using the MDC incorporation assay (Figure 4D). These two findings support the conclusion from the phylogenetic analysis that culicines lack an orthologue of the plug forming TGase and strengthen the correlation between the presence of *AGAP009099* in *An. gambiae* and plug formation in this species.

## Discussion

We have identified the molecular composition, mechanism of formation, and function of the mating plug of *An. gambiae*. Our MS analysis identified 15 MAG-expressed proteins that are transferred to females as part of the mating plug. Two of these proteins, the MAG-specific TGase AGAP009099 and its glutamine-rich substrate Plugin, are responsible for the coagulation of the liquid secretions of the MAGs into a solid mass. Some of the other MAG-proteins from the plug, particularly the three small Acp-like proteins AGAP009362, AGAP009370, and AGAP012830, could represent important modulators of female behavioural responses to copulation, such as a reduced receptivity to further mating and induced oviposition [9]. To our knowledge, these are the first proteins transferred to females during mating that have been identified in *Anopheles*. Further studies will clarify the role of these proteins in modulating female reproductive biology and possibly in sperm function. The identification of a number of female proteins, mainly proteases, associated with the mating plug suggests a direct interaction between male and female proteins that may be important for plug processing. Indeed two of the female proteases identified on the plug (AGAP005194 and AGAP005195) were shown previously to be expressed exclusively in the atrium of virgin females and were considerably downregulated at 24 h after mating [24]. This transcriptional modulation is entirely compatible with a role of these enzymes in plug digestion, which is mostly completed in the female atrium by 24 h post-mating.

Mating plugs are found in a wide assortment of vertebrate and invertebrate species, and many hypotheses have been advanced to explain their function. However, in the vast majority of taxa, empirical evidence for a specific role of the plug in mating remains elusive [14]. Perhaps the most common presumption is that plugs act as barriers to re-insemination. The high levels of monogamy observed in wild mosquito populations are thought to be enforced, at least over the short term, by the presence of a mating plug [15]. We have shown that the plug provides little defence against the storage of sperm from subsequent males. This is consistent with the observed mating behaviour of *An. gambiae*. In this generally monoandrous species, virgin females enter a swarm of males, mate, and leave the swarm while still in copula [28],[29]. It is unlikely that a female would re-enter the swarm (and indeed double plugs are almost never observed in the field [12],[30]), and therefore there would be very little selective pressure for a plug that acts as a physical block.

Instead, we have demonstrated that the plug plays an important role in the reproductive biology of *An. gambiae*. By manipulating the expression of the MAG-specific TGase, we have prevented plug formation and transfer, resulting in the incomplete storage of sperm by the female. The presence of a plug in the early post-copulatory hours may be needed to facilitate sperm retention in the sperm storage organ until motility is acquired. Indeed the sperm of *An. gambiae* are deposited in a immotile state directly into the spermatheca, immediately followed by plug transfer [12],[31], and become motile only >17 h after copulation [32]. In support of this hypothesis, we observed sperm in the atria of females that did not receive a plug, strongly suggesting that they had been transferred but had leaked out of the spermatheca. Interestingly, a role for the mating plug in sperm storage was dismissed by some earlier researchers [12]. Using the forced copulation technique for mating, females could be inseminated even when they did not receive a plug [33]. However, the forced copulation required the female to be anaesthetized during mating. One possible explanation for these earlier results is that female activity is required for sperm loss. Sperm backflow from the spermatheca into the atrium occurring in plug-less matings may be a passive consequence of female movement, or—more intriguingly—females may use the mating plug as a male “quality check,” actively ejecting sperm from males that fail to transfer it. As the spermathecae of a small number of females that did not receive a plug contained observable sperm, future investigations of the relative numbers of sperm stored by these females could shed light on this issue. An alternative or additional explanation for the observed lack of sperm in the storage organs of females mated with plug-less males is that the formation of a complete mating plug in the male reproductive tract may be important for the correct delivery of sperm to the female. Although sperm are directly deposited in the spermatheca prior to plug transfer, it cannot be ruled out at this stage that coagulation of seminal fluids within the MAGs may play a role in the successful completion of the transfer process.

The expansion of the TGase family in mosquitoes, and the acquisition of a function in seminal coagulation, underlies the ability of *An. gambiae* to form mating plugs. The presence of multiple TGases has not been previously reported in insects, but is common in vertebrates, and nine have been characterized in mammals [27]. These proteins fulfil numerous functions including seminal coagulation [34], which is achieved by the cross-linking of glutamine-rich substrates such as semenogelins and seminal vesicle secretory proteins by the prostate-specific TGase TGM4 [34]–[36]. Thus, mosquitoes and mammals have independently evolved highly similar systems of semen coagulation. The convergent evolution of similar systems for plug formation in mosquitoes and in mammals is made all the more remarkable by the fact that other organisms have developed very different TGase-independent mechanisms to achieve similar results [37]–[39]. TGase-mediated cross-linking of plug proteins seems to be finely controlled as the plug-forming TGase and its major substrate (i.e., Plugin) are expressed in two different compartments of the MAGs (Figure 2C, 2D, Figure 3D). These two proteins may be brought together in the aedegus during copulation by contraction of the muscle cells surrounding the MAGs, and a concomitant release of calcium from intracellular stores could cause the activation of the secreted TGase.

Our findings reveal a crucial role of the mating plug in mosquito reproductive biology and identify this important structure as a potential target for the manipulation of mosquito fertility. This discovery was only possible because we first identified the molecular composition and mechanism of formation of the plug. Understanding the basic genetics underlying mating biology is an essential starting point for developing new tools that target mosquito reproduction and may influence the design of novel vector control strategies currently under development. The proteins identified in this study will not only provide a powerful basis for understanding other processes that regulate mosquito fertility, but will also allow comparative studies of reproduction in other organisms. Indeed, given the remarkable similarity between mechanisms of seminal coagulation in mosquitoes and mammals, our results can inform studies of mammalian, including human, reproduction.

## Materials and Methods

### Mosquito Procedures

Mosquitoes from a laboratory colony of the G3 strain were separated by sex as pupae and raised in cages supplied with sucrose *ad libitum*. Matings were performed as described previously [24].

### Mass Spectrometry

Reproductive tissues were dissected from virgin males (MAGs), virgin females (atria), or recently mated females (mating plugs); washed in fresh PBS; and stored on ice in 20 µl of a 5% (v/v) acetic acid solution. The overall digest, chromatographic, and MS strategies used have been described previously [19]–[23]. Briefly, the supernatant was applied to SDS precast NuPAGE gels and following electrophoresis, Coomassie-stained. Bands were excised, destained, reduced, and alkylated with iodoacetic acid prior to proteolytic digestion with trypsin. After extraction the peptide mixtures were analysed by on-line nanoLC-MS and MS/MS using Q-TOF technology on Q-TOF and Q-Star instruments and by Mascot search of the MSDB/NCBI and *An. gambiae* database initially, then using *An. gambiae* predicted proteome ReAnoXcel [40] supplemented with the latest Ensembl and SNAP protein predictions. Identified peptides were individually blasted against the translated genome, and gene models corresponding to the identified genomic regions were developed using *ab initio* predictions informed by available ESTs, microarray data, and manual models submitted to Vectorbase. The genomic location of each gene model is provided in Table S3. The source of proteins identified in the plug was confirmed by RT-PCR performed using cDNAs from MAGs, testes, the rest of the male body, and virgin non-bloodfed females.

### MDC-Incorporation Assay

MAGs were dissected from 4-d-old virgin males, homogenized with a micropestle in either TGase “+” buffer (50 mM Tris pH 7.6, 1 mM DTT, 5 mM CaCl_2_) or TGase “−” buffer (TGase “+” buffer with 250 mM EDTA and 0.3 mM dGTP) and frozen/thawed in dry ice three times before the addition of 5 mM MDC. Samples were incubated at 37°C for 60 min, vortexed briefly, and spun down 10 min at 13,000 rpm. Proteins in the supernatant were separated by SDS-PAGE and visualized under UV illumination using an LAS-3000 imaging system (FujiFilm). Plugin localization was subsequently tested by Western blot. Total protein loaded on gels was visualized using SimplyBlue SafeStain (Invitrogen).

### Polyclonal Antibodies

Affinity-purified polyclonal antibodies against Plugin and AGAP009099 were raised in rabbits against peptide epitopes (Plugin: NEHRDPQNHQLPSSC; AGAP009099: CGSRYTDPMEKKYES) by a commercial supplier (GenScript Corp., Piscataway, NJ).

### Western Blots

Tissues were homogenized in 20 µl PBS containing a protease inhibitor cocktail (Complete Mini, Roche) and frozen/thawed three times on dry ice. Samples were centrifuged at 13,000 rpm for 15 min at 4°C. The supernatant was heated at 70°C for 10 min and applied to precast NuPAGE (Invitrogen) gels under reducing conditions according to the manufacturer's instructions. Proteins were transferred to a nitrocellulose membrane (under reducing conditions) using the XCell II Blot module (Invitrogen). Blots were immunostained using standard protocols using the following primary antibody titres: anti-Plugin: 0.59 µg/ml; anti-9099: 0.96 µg/ml; and anti-β-actin (1∶1000 dilution of ab8229; Abcam, Cambridge, MA). HRP-conjugated secondary antibodies (Santa Cruz Biotechnologies: sc-2030 and sc-2314) were used at a dilution of 1∶10,000. Bands were visualized using ECL Western blotting detection reagents (GE Heatlhcare) on an LAS-3000 imaging system (FujiFilm).

### ELISA

Individual MAGs from males injected with *ds9099* or *dsLacZ*, were placed in a 110 µl PBS containing a protease inhibitor cocktail (Complete Mini, Roche), homogenized in an ultrasonic bath for 10 min, frozen/thawed on dry ice three times, and centrifuged at 13,000 rpm for 15 min at 4°C. Duplicate 50 µl aliquots of the supernatant were loaded into separate wells of a flat-bottomed 96-well plate and incubated overnight at 4°C. A standard curve was prepared from the MAGs of uninjected males with a series of six 2-fold dilutions. ELISAs were carried out essentially as described previously [41]. Anti-9099 was used at a concentration of 0.96 µg/ml and the secondary antibody, sc-2314, at a dilution of 1∶2,000.

### Immunostaining and Confocal Analysis

MAGs from 3–4-d-old males were dissected on ice, fixed in 4% formaldehyde, washed in PBS, bleached with 2% hydrogen peroxide to minimize autofluorescence, washed in PBS, then blocked and permeabilized in PBS with 1% BSA and 0.03% Triton X-100. Samples were incubated in either 2 µg/ml anti-Plugin or 3 µg/ml anti-9099 in blocking buffer, then a 1∶1,000 dilution of anti-rabbit Cy3 followed by a 1∶250 dilution of Alexa Fluor 488 phalloidin (Invitrogen) to stain actin. Tissues were then mounted in DAPI-containing Vectashield medium (Vector Laboratories, Inc.) and visualized using a Leica SP5 inverted confocal microscope. Stacks were generated using 19 consecutive 0.5 µm optical sections.

### RNA Interference

A 481 bp region of *AGAP009099* was amplified from MAG cDNA using the primers (FWD: 5′-GAGCGGTCGTGGTCGATAGTAAG-3′ and REV: 5′-CCCTCGTAGTTGTTGCTCCAGTT-3′) and cloned into pLL10 [42]. This purified linearized plasmid was used to make *ds9099*, and pLL100 for the synthesis of *dsLacZ* (dsRNA targeting the bacterial *LacZ* transcript, which is not present in mosquitoes), following established protocols [42],[43]. Males were sexed as pupae and injected with 69 nl of dsRNA (3 µg/µl) within 24 h of eclosion. Surviving males were allowed to mate with 5–6-d-old virgin females 4–5 d after injection. Mated females were immediately dissected to visually ascertain the presence of a mating plug in the atrium and/or sperm in the spermatheca. Males were dissected and their MAGs used for qRT-PCR analysis of RNAi-induced knockdown, or for ELISA.

### qRT-PCR

RNA extraction, cDNA synthesis, and SYBR-green based qRT-PCR was performed as described previously [24] using the primers listed in Table S1. The ribosomal protein gene *RpL19* was used for normalization in *An. gambiae* (*AGAP004422*), using previously described primers [24].

### Remating Assay

Wild-type 4-d-old virgin females were placed in a cage containing approximately 250 wild-type males. Copulating pairs were captured as described previously [24], the males removed, and the females introduced into a cage containing approximately 250 males homozygous for the transgene *dsRed* (FC, unpublished data). Females mating for a second time were recaptured and placed in a cage without males for 48 h. After this period, females were dissected in PBS, and individual spermathecae were placed in 23 µl of grinding buffer (80 mM NaCl, 8.5 mM EDTA, 24 mM Tris [pH 7.5], 0.5% SDS, and 5.5% sucrose). Samples were placed in an ultrasonic water bath for 10 min or until each spermatheca was ruptured. Three µl of 0.01 M Proteinase K was added to each tube, and samples were heated at 37°C for 15 min, then 95°C for 10 min. Samples were analyzed by SYBR green-based qPCR using 5 µl of undiluted spermathecal DNA. Y-specific primers (Table S1) were designed within the Y-specific region of the chimeric *An. gambiae* scaffold AAAB01008227 amplified by Kryzywinski et al. [44] using the primer pair 128125I. These primers were tested on multiple male and female genomic DNA extractions and only produced a product in males (two copies per Y-chromosome, unpublished data). In both cases, matings were completed in the space of 60 min. Females were rested for 45 min between the two matings.

### Phylogenetics

The selected amino acid sequences were subjected to multiple alignments using the Clustal W (http://www.ebi.ac.uk/Tools/clustalw/) and Clustal X (1.83) algorithms. A phylogenetic tree was constructed by the neighbour joining method using p-distance estimates, tested by the interior-branch test, and displayed using TreeView (1.6.6) software. Reliability of each node was assessed with 1,000 bootstrap replications. The genomic locations of the TGase genes encoding the proteins used in the tree, as well as Plugin, are reported in Table S2.

## Supporting Information

Figure S1 **The Plugin protein.** (A) An example of the nano LC-MS data created in this study. The total ion current trace across the nanoLC chromatogram over 90 min in the analysis of SDS PAGE band 13 of a MAG sample (gel Mr ∼70 kD), highlighting the region at 42.4 min for subsequent on-line MS/MS analysis. (B) Elucidation of the true N terminus of the mature Plugin protein: The data-dependent acquisition of an MS/MS spectrum of a doubly charged quasimolecular ion, m/z 809.62+, elution time 42.4 min, sequenced as VPL/IYGGVDQQFGL/IPK. A weaker signal at m/z 859.12+ from the dataset is assigned as the same sequence with an additional N-terminal Valine. The predicted signal sequence, residues 1–16, for the Plugin protein would suggest an N terminus beginning at Ala-17 of Ala-Val-Val-Pro for the expressed product. The experimental data show that the major processing event leads to an N terminus beginning at Val-19 for the Plugin protein. (C) The complete amino acid sequence of Plugin. The Plugin sequence was determined using a combination of MS and molecular biological methods. The 5′ end was elucidated using the FirstChoice RLM-RACE Kit (Ambion) according to the manufacturer's instructions, starting with primers against the genomic region matching initial peptides identified by mass spec analysis which provided over 50% (underlined) sequence coverage (outer primer: 5′-TGCGCTAGTTGCTGCTTTTGGT-3′; inner primer: 5′-GCTGCTCCTGCTCCTTGATCCT-3′). The 3′ end of the gene was identified by designing primers against *ab initio* predictions in the region and sequencing the resulting RT-PCR products until an in-frame stop codon was identified. Putative transglutamination sites are highlighted in red. The sequence from residues 19–234 is predicted to be intrinsically disordered.
(0.34 MB PDF)

Table S1 **Primers used for quantitative PCR.** Primers against Plugin and TGases were used for qRT-PCR. Ribosomal genes were used as controls for normalization. For *An. gambiae*, we used primers against *RpL19* (AGAP004422). For *A. aegypti* and *C. quinquefasciatus*, we used a single set of primers against *RpS7* (AAEL009496 and CPIJ006763, respectively), which perfectly matched the sequences in both species. Primers against the Y-chromosome of *An. gambiae* and the *dsRed*-containing transgenic cassette were used to quantify relative sperm numbers by standard quantitative PCR in the remating experiment.
(0.09 MB PDF)

Table S2 **Genomic locations of Plugin and TGase gene predictions.** VectorBase IDs that refer to incomplete or otherwise incorrect gene predictions are indicated by an asterisk. The genomic positions of our corrected predictions are indicated by the chromosome arm (CA, orientation in parentheses) or the super contig (SC) number, as well as the specific location. Only Plugin and AGAP009099 were fully sequenced. The reported locations of other genes refer only to *ab initio* predictions. *Ab initio* predictions for mosquito TGase genes were determined using BLAST searches using known TGases from other species and scanning the resulting genomic regions with *ab initio* gene prediction software (Fgenesh, Genescan, and Augustus). Each consensus sequence was analyzed for conserved domains using PFAM 22.0 and SMART 5.1 to ensure it contained the three canonical TGase domains.
(0.08 MB PDF)

Table S3 **Summary of MS/MS results.** Proteins identified by MS/MS analysis of mating plugs (plug), male accessory glands (MAGs), and virgin female atria (atrium) are listed on separate spreadsheets. Protein identifiers are the same as those listed in Figure 1, and the locations refer to the chromosomal arm followed by the exact position of the predicted proteins on the AgamP3 genome assembly. The total number of residues in each protein prediction (the signal peptide has been removed where appropriate) is described in the column “protein size” followed by the number of residues identified in the MS/MS analysis (“identified”). These values were used to calculate the percentage coverage. “Total hits” refers to the complete number of peptide matches to each protein, including redundant matches found in multiple gel bands or in more than one replicate sample. “N-R hits” refers to the number of non-redundant peptide matches across all gel bands and replicates. The expected mass of each predicted protein (signal peptides removed where appropriate) was calculated using Protein Calculator (http://www.scripps.edu/~cdputnam/protcalc.html). The observed mass refers to the approximate distance migrated by the gel band in which the peptides were identified. When peptides matching a single protein were identified in multiple bands, approximate sizes are separated by semi-colons. For peptides found in many bands, only the range is provided. Mascot protein assignments were based on nano-LC MS/MS data with ion scores>60 (*p*<0.05) together with multiple matches and total protein scores, with outliers accepted subject to visual inspection and sequence verification. Inclusion of assignments in the table was then further subject to confirmation by RT-PCR.
(0.07 MB XLS)

## Abbreviations

dsRNA: double stranded RNA
MAGs: male accessory glands
MDC: monodansylcadaverine
MS: mass spectrometric
RT-PCR: reverse transcription PCR
TGase: transglutaminase
