# Supplementary material for: Transglutaminase-Mediated Semen Coagulation Controls Sperm Storage in the Malaria Mosquito
Source: PLoS Biol. 2009 Dec 22;7(12):e1000272. doi: 10.1371/journal.pbio.1000272 (PMC2785878; doi:10.1371/journal.pbio.1000272)
Supplement: Figure S1 — The Plugin protein. (A) An example of the nano LC-MS data created in this study. The total ion current trace across the nanoLC chromatogram over 90 min in the analysis of SDS PAGE band 13 of a MAG sample (gel Mr ∼70 kD), highlighting the region at 42.4 min for subsequent on-line MS/MS analysis. (B) Elucidation of the true N terminus of the mature Plugin protein: The data-dependent acquisition of an MS/MS spectrum of a doubly charged quasimolecular ion, m/z 809.62+, elution time 42.4 min, sequenced as VPL/IYGGVDQQFGL/IPK. A weaker signal at m/z 859.12+ from the dataset is assigned as the same sequence with an additional N-terminal Valine. The predicted signal sequence, residues 1–16, for the Plugin protein would suggest an N terminus beginning at Ala-17 of Ala-Val-Val-Pro for the expressed product. The experimental data show that the major processing event leads to an N terminus beginning at Val-19 for the Plugin protein. (C) The complete amino acid sequence of Plugin. The Plugin sequence was determined using a combination of MS and molecular biological methods. The 5′ end was elucidated using the FirstChoice RLM-RACE Kit (Ambion) according to the manufacturer's instructions, starting with primers against the genomic region matching initial peptides identified by mass spec analysis which provided over 50% (underlined) sequence coverage (outer primer: 5′-TGCGCTAGTTGCTGCTTTTGGT-3′; inner primer: 5′-GCTGCTCCTGCTCCTTGATCCT-3′). The 3′ end of the gene was identified by designing primers against ab initio predictions in the region and sequencing the resulting RT-PCR products until an in-frame stop codon was identified. Putative transglutamination sites are highlighted in red. The sequence from residues 19–234 is predicted to be intrinsically disordered. (0.34 MB PDF) [file pbio.1000272.s001.pdf]

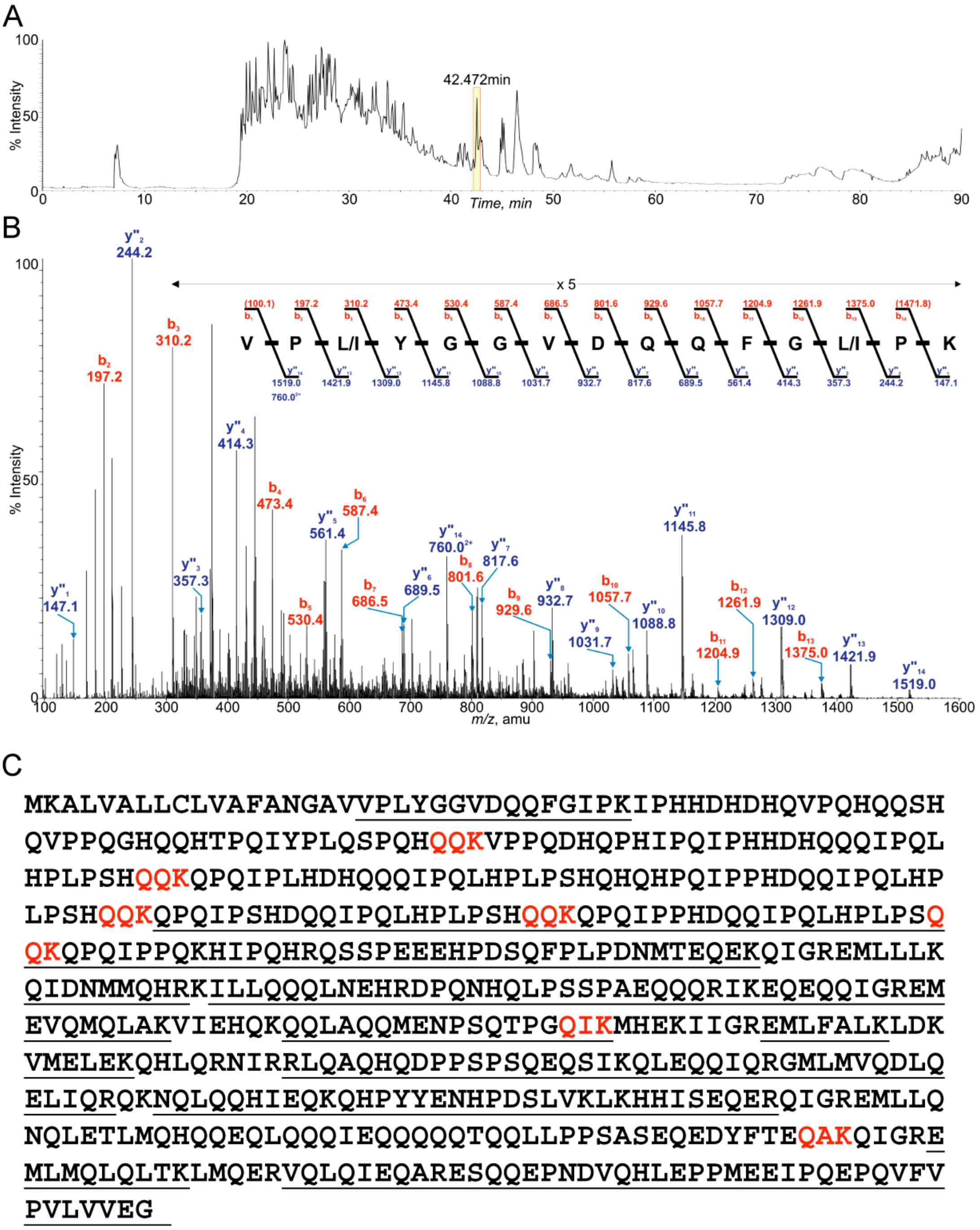


**Supplementary Fig. 1**. **The Plugin protein. A.** An example of the nano LC-MS data created in this study. The total ion current trace across the nanoLC chromatogram over 90mins in the analysis of SDS PAGE band 13 of a MAG sample (gel Mr ~70kD), highlighting the region at 42.4 mins for subsequent on-line MS/MS analysis. **B.** Elucidation of the true N-terminus of the

mature Plugin protein: The data-dependent acquisition of an MS/MS spectrum of a doubly charged quasimolecular ion, m/z $809.6^{2+}$, elution time 42.4mins mins, sequenced as VPL/IYGGVDQQFGL/IPK. A weaker signal at m/z $859.1^{2+}$ from the data set is assigned as the same sequence with an additional N-terminal Valine. The predicted signal sequence, residues 1-16, for the Plugin protein would suggest an N-terminus beginning at Ala-17 of Ala-Val-Val Pro....... for the expressed product. The experimental data show that the major processing event leads to an N-terminus beginning at Val-19 for the Plugin protein. **C.** The complete amino acid sequence of Plugin. The Plugin sequence was determined using a combination of mass spectrometric and molecular biological methods. The 5' end was elucidated using the FirstChoice RLM-RACE Kit (Ambion) according to the manufacturers instructions, starting with primers against the genomic region matching initial peptides identified by mass spec analysis which provided over 50% (underlined) sequence coverage (outer primer: 5'-TGCGCTAGTTGCTGCTTTTGGT-3'; inner primer: 5'-GCTGCTCCTGCTCCTTGATCCT-3'). The 3'end of the gene was identified by designing primers against *ab initio* predictions in the region, and sequencing the resulting RT-PCR products until an in-frame stop codon was identified. Putative transglutamination sites are highlighted in red. The sequence from residues 19-234 is predicted to be intrinsically disordered.
