# Supplementary material for: Transglutaminase-Mediated Semen Coagulation Controls Sperm Storage in the Malaria Mosquito
Source: PLoS Biol. 2009 Dec 22;7(12):e1000272. doi: 10.1371/journal.pbio.1000272 (PMC2785878; doi:10.1371/journal.pbio.1000272)
Supplement: Table S1 — Primers used for quantitative PCR. Primers against Plugin and TGases were used for qRT-PCR. Ribosomal genes were used as controls for normalization. For An. gambiae, we used primers against RpL19 (AGAP004422). For A. aegypti and C. quinquefasciatus, we used a single set of primers against RpS7 (AAEL009496 and CPIJ006763, respectively), which perfectly matched the sequences in both species. Primers against the Y-chromosome of An. gambiae and the dsRed-containing transgenic cassette were used to quantify relative sperm numbers by standard quantitative PCR in the remating experiment. (0.09 MB PDF) [file pbio.1000272.s002.pdf]

Gene ID	Sequence (5'-3')	Conc (nM)	Amplicon length (bp)
PLUGIN	TGATTCAACCGTAGACATGAAGG	300	87
	CCACCATACAACGGAACGAC	300	
AGAP009098	CACCATTCCGATTGAGTTTGTT	300	88
	CAGTTGAGTGCTTCCCTTGA	900	
AGAP009099	CAACCATGTCTACCACCTCTACC	300	150
	ATCCTTTATCGCACTGTTCGTC	300	
AGAP009100	AGAGTTATTCGCTTCCGCAG	300	87
	GGTGTGCTTTCTCTTCCATGT	900	
AEDES 1	CAGTGGATTTTTACCTACTGTTCAATC	300	113
	TCGCCAAATCATTGTCGTATC	900	
AEDES 2	TCGTTTTATTTGCTGTTCAATCC	300	147
	GAATTTCCACACCGAAGGTC	900	
CULEX 1	TACTTGCTGTTTAATCCGTGGTG	300	100
	TCCAGATCAAAGTGGTATCGTTC	300	
CULEX 2	TTGTCCCCGTTGATGATGT	300	116
	GTACGATGAGCCTCTCCATTCT	300	
RpS7	ATGGTTTTCGGATCAAAGGTG	300	111
	CGGCTTCAGGTCCGAGTT	300	
Y-specific	GGATCTGGCCAAGAGGAGTA	300	148
	CCCAACCAAGGTACTCTAACG	300	
DsRed	ATGGTGCGCTCCTCCAAGAACG	300	146
	ACCTTCAGCTTCACGGTGTTGTGG	300	

Supplementary Table 1. Primers used for quantitative PCR. Primers against Plugin and TGases were used for qRT-PCR. Ribosomal genes were used as controls for normalization. For *An. gambiae*, we used against *RpL19* (AGAP004422). For *A. aegypti* and *C. quinquefasciatus*, we used a single set of primers against *RpS7* (AAEL009496 and CPIJ006763, respectively) which perfectly matched the sequences in both species. Primers against the Y-chromosome of *An. gambiae* and the *dsRed*-containing transgenic cassette were used to quantify relative sperm numbers by standard quantitative PCR in the remating experiment.
