# Supplementary material for: Transglutaminase-Mediated Semen Coagulation Controls Sperm Storage in the Malaria Mosquito
Source: PLoS Biol. 2009 Dec 22;7(12):e1000272. doi: 10.1371/journal.pbio.1000272 (PMC2785878; doi:10.1371/journal.pbio.1000272)
Supplement: Table S2 — Genomic locations of Plugin and TGase gene predictions. VectorBase IDs that refer to incomplete or otherwise incorrect gene predictions are indicated by an asterisk. The genomic positions of our corrected predictions are indicated by the chromosome arm (CA, orientation in parentheses) or the super contig (SC) number, as well as the specific location. Only Plugin and AGAP009099 were fully sequenced. The reported locations of other genes refer only to ab initio predictions. Ab initio predictions for mosquito TGase genes were determined using BLAST searches using known TGases from other species and scanning the resulting genomic regions with ab initio gene prediction software (Fgenesh, Genescan, and Augustus). Each consensus sequence was analyzed for conserved domains using PFAM 22.0 and SMART 5.1 to ensure it contained the three canonical TGase domains. (0.08 MB PDF) [file pbio.1000272.s003.pdf]

| Gene | VectorBase ID | CA/SC | Location |
|---|---|---|---|
| Plugin | AGAP009368* | 3R (–) | 31,812,524 – 31,814,708 |
| AGAP009098 | AGAP009098* | 3R (–) | 25,583,824 – 25,586,349 |
| AGAP009099 | AGAP009099* | 3R (+) | 25,588,861 – 25,591,488 |
| AGAP009100 | AGAP009100 | 3R (+) | 25,596,779 – 25,599,732 |
| AEDES 1 | AAEL006978* | 1.230 | 224,090 – 229,150 |
| AEDES 2 | AAEL006978* | 1.230 | 180,069 – 184,614 |
| CULEX 1 | CPIJ003524 | 3.48 | 900,874 - 903,335 |
| CULEX 2 | CPIJ003523* | 3.48 | 886,757 – 889,084 |

**Supplementary Table 2. Genomic locations of Plugin and TGase gene predictions.** VectorBase IDs that refer to incomplete or otherwise incorrect gene predictions are indicated by an asterisk. The genomic positions of our corrected predictions are indicated by the chromosome arm (CA, orientation in parentheses) or the super contig (SC) number, as well as the specific location. Only Plugin and AGAP009099 were fully sequenced. The reported locations of other genes refer only to *ab initio* predictions. *Ab initio* predictions for mosquito TGase genes were determined using BLAST searches using known TGases from other species and scanning the resulting genomic regions with *ab initio* gene prediction software (Fgenesh, Genescan, and Augustus). Each consensus sequence was analyzed for conserved domains using PFAM 22.0 and SMART 5.1 to ensure it contained the three canonical TGase domains.
